# Pacific bluefin tuna (*Thunnus orientalis*) bycatch in the Atlantic Ocean

**DOI:** 10.1111/jfb.70463

**Published:** 2026-04-21

**Authors:** Yohei Tsukahara, Atsushi Tawa, Masashi Sekino, Kenji Nohara, Nobuaki Suzuki

**Affiliations:** ^1^ Highly Migratory Species Division Fisheries Resources Institute, Japan Fisheries Research and Education Agency Yokohama Kanagawa Japan; ^2^ Bioinformatics and Biosciences Division Fisheries Resources Institute, Japan Fisheries Research and Education Agency Yokohama Kanagawa Japan; ^3^ Department of Marine Biology School of Marine Science and Technology, Tokai University Shimizu Shizuoka Japan

**Keywords:** bycatch, longline fishery, mitochondrial genes, morphology, single‐nucleotide polymorphisms, species identification

## Abstract

A large tuna was hooked by a Japanese longline fishery in the high seas near the Republic of Namibia. This fishing ground is mainly utilized for *Thunnus albacares*, *Thunnus obesus* and *Thunnus alalunga* by Japanese tuna longliners. Because the tuna was larger than the common size of these species, the specimen was sampled for genetic species identification and morphological measurements. The fish, which weighed 107 kg without gills and gut, was unexpectedly identified as *Thunnus orientalis* by a thorough analysis of morphology, mitochondrial genes and nuclear single‐nucleotide polymorphisms.

Japanese longline vessels for tuna operate in enormously wide areas of the ocean, including the Pacific, Indian and Atlantic Oceans. In the Atlantic Ocean, *Thunnus thynnus* (Linnaeus, 1758) (Atlantic bluefin tuna) is targeted in the temperate and subarctic area of the northern hemisphere, while longliners target tropical tuna species such as *Thunnus albacares* (Bonnaterre, 1788) (Yellowfin tuna), *Thunnus obesus* (Lowe, 1839) (Bigeye tuna) and *Thunnus alalunga* (Bonnaterre, 1788) (Albacore) around tropical and temperate areas in the southern hemisphere. On 21 June 2024, a vessel operating in the high seas of the Atlantic Ocean in the vicinity of the Republic of Namibia (26° S and 10° E) caught more than 286 *T. alalunga* and some other fish, along with a fish that weighed 107 kg without gills or internal organs (Figure 1a). It was seemingly larger than the typical size of *T. obesus* and *T. albacares*, which is largely less than 100 kg in round weight, and was consequently reported as a bycatch of bluefin tuna.

**FIGURE 1:**
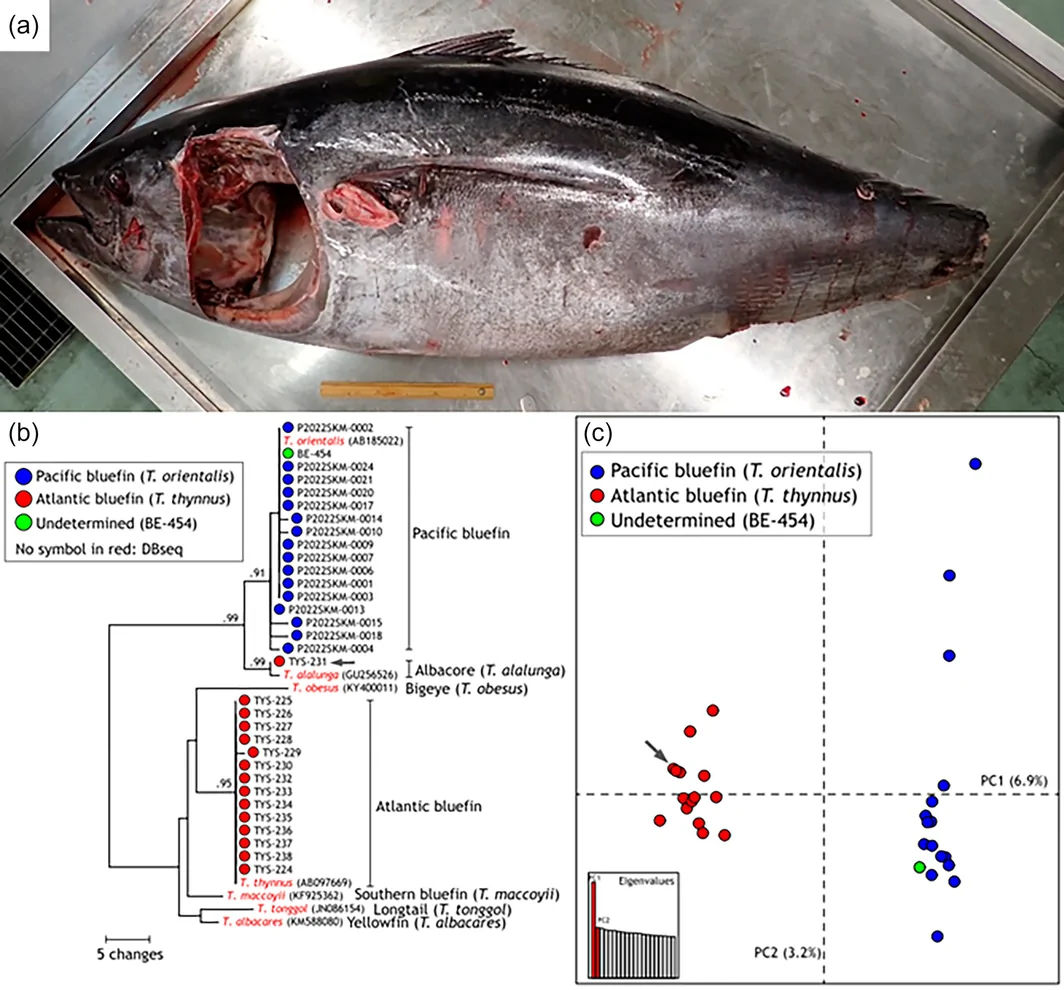
(a) Picture of the captured fish with 30 cm scale, (b) maximum parsimony tree showing the separation between *Thunnus orientalis* and *Thunnus thynnus* based on the nucleotide sequences of mitochondrial genes, (c) principal component analysis (PCA) plotting of individuals based on restricted site‐associated DNA (RAD)‐derived single‐nucleotide polymorphism (SNP) genotypes. Arrows to *T. thynnus* specimen in (b) and (c) indicate a *Thunnus alalunga*‐like mitochondrial haplotype.

According to the official catch record of the International Commission for the Conservation of Atlantic Tunas (ICCAT) since 1950, however, previous to this observation, no catch report existed for Atlantic bluefin tuna in the area south of 20° S and east of 25° E of the Atlantic Ocean, which is named as ‘BF66’ under ICCAT management. Due to the uniqueness of this fish, we obtained a piece of muscle tissue and collected as much morphological data as possible to identify its species. For convenience, we refer to this individual hereafter as ‘BE‐454’.

Eight species of the genus *Thunnus* are currently recognized worldwide (Chow & Kishino, 1995; Collette, 1999; Iwai et al., 1965). Of these, five can exceed 100 kg in body weight: *Thunnus orientalis* (Temminck and Schlegel, 1844) (Pacific bluefin tuna), *T. thynnus*, *T. albacares*, *T. obesus and Thunnus maccoyii* (Castelnau, 1872) (Southern bluefin tuna). In general, these five species can be morphologically identified by several traits such as body colour, fin colour, pectoral fin length, number of gill rakers and liver shape (Iwai et al., 1965). However, because several body parts, such as the gills, internal organs, pectoral fins and caudal fin were removed from BE‐454 on board, as shown in Figure 1a, these features were unavailable for its species identification.

Morphological measurements with a large Vernier calliper were performed at 10 measurable points (Table 1), following Iwai et al. (1965) and Nakabo (2013). Additionally, we measured body depth at the first dorsal spine of the dorsal fin and anus. The bulging of the dorsal wall into the abdominal cavity was also visually examined, according to Gibbs and Collette (1967). The length from the tip of the snout to the tailward end of the body was 155.5 cm. The values of the other morphological features are summarized in Table 1. The tail was cut off near the fifth finlet on the ventral side; hence, the missing tail was likely to be 20–30 cm long, resulting in an estimated fork length of approximately 175.5–185.5 cm. The ratio of eye diameter (ED) to maxillary length was 37.2%, substantially smaller than the common ratio for *T. obesus*, which is approximately 50% (Ochiai & Tanaka, 1986). The ratio of ED to head length was 14.0%, consistent with the common value for *T. orientalis* and *T. thynnus* (12.5%–16.7%) (Ochiai & Tanaka, 1986).

**TABLE 1:** Results of morphological measurement.

Part	Value (cm)
Head length	55.0
Preanal length	112.5
Predorsal fin length	59.0
Maximum body depth	48.1
Snout length	19.0
Eye diameter	7.7
Maxillary length	20.7
Interorbital width	23.0
Body depth at first dorsal spine	47.7
Body depth at anal	36.9

No noticeable bulging of the dorsal wall was observed in the abdominal cavity. Because the bulging of the dorsal wall is a key trait of *T. thynnus* and *T. maccoyii* (Gibbs & Collette, 1967), BE‐454 was not considered to be either of these two species. Based on these observations, the external morphology was most likely to be *T. orientalis*, although no clear evidence exists to exclude *T. albacares* as a possibility. Generally, *T. orientalis* and *T. albacares* are distinguishable by several traits, such as the length of the pectoral and second dorsal sides, whereas these traits are unavailable for BE‐454. Nevertheless, the body colour was black with no yellow bands on either side. ED was small relative to head length. Body depth was tall. All of these traits, although not conclusive, support the idea that BE‐454 is *T. orientalis* rather than *T. albacares*.

DNA barcoding based on mitochondrial DNA (mtDNA) is commonly used for species identification of *Thunnus* (Amaral et al., 2017; Terio et al., 2010). However, *T. alalunga*‐like mitochondrial haplotypes have been observed in both *T. orientalis* and *T. thynnus*, most likely because of introgression and/or incomplete lineage sorting (ILS). *T. orientalis*‐like mitochondrial haplotypes in *T. thynnus* and vice versa have also been reported, possibly because of the same phenomenon (Alvarado Bremer et al., 2005; Chow et al., 2006; Chow & Kishino, 1995). These observations suggest that the use of mtDNA alone may lead to erroneous species identification. Therefore, we examined both the mtDNA and nuclear DNA (nDNA) of BE‐454, along with reference specimens of *T. orientalis* and *T. thynnus*.

The specimens used as references were collected from the common fishing grounds of *T. orientalis* (*N* = 16) and *T. thynnus* (*N* = 15). *T. orientalis* was caught by purse seine in the Sea of Japan, whereas *T. thynnus* was caught by longline in the North Atlantic Ocean. In total, 32 specimens, that is, BE‐454 and 31 references, were used for mtDNA and nDNA analyses. Additionally, seven complete mitogenome sequences in the EMBL/DDBJ/GenBank database (hereafter referred to as ‘DBseq’) were utilized for mtDNA‐based DNA barcoding: *T. orientalis* (acc., AB185022), *T. thynnus* (AB097669), *T. maccoyii* (KF925362), *T. alalunga* (GU256526), *T. obesus* (KY400011), *Thunnus tonggol* (JN086154) and *T. albacares* (KM588080).

For mtDNA analysis, partial adenosine triphosphate synthase subunit 6 (*ATP6*) and cytochrome c oxidase subunit III (*COX3*) genes were amplified via polymerase chain reaction (PCR) with a PCR primer set described by Chow and Kishino (1995), whereas a primer in Palumbi et al. (2002) (16sar‐L and 16sbr‐H) was utilized for the amplification of the partial 16S rRNA (*16S*) gene. PCR protocols are described in Supporting Information 1. The method described by Sekino and Yamashita (2013) was followed for post‐PCR enzymatic purification of amplicons, Sanger sequencing, trimming and alignment. The resulting sequences, with no insertion/deletion polymorphisms across the genes among the 32 specimens and seven DBseqs, were concatenated (1343 bases; *ATP6* + *COX3*, 791 bases; *16S*, 552 bases) (see Supporting Information 2 for accession numbers). Using these concatenated sequences, a maximum parsimony (MP) tree was reconstructed based on a subtree‐pruning‐regrafting algorithm with search level 3 (MEGA version 6.06; Tamura et al., 2013), in which node credibility was evaluated using 1000 bootstrap replications.

For nDNA analysis, single‐nucleotide polymorphisms (SNPs) were identified based on restricted site‐associated DNA (RAD) sequencing (Baird et al., 2008) using the restriction enzyme *Sbf* I (SbfI‐HF; New England Biolabs). The protocol for RAD library construction was previously described by Sekino et al. (2016) (Supporting Information 1 therein). The library, along with PhiX control v3 (Illumina) at a molar concentration ratio of 15%, was sequenced for up to 85 bases (single path) on NextSeq 500 combined with NextSeq 500 High Output v2.5 Kit 75 Cycles (Illumina). Subsequent demultiplexing and quality filtering of raw reads, mapping onto a reference genome sequence (*T. thynnus*; GCF_963924715.1), SNP calling and SNP filtering are described in detail in Supporting Information 3. The demultiplexed raw sequence reads (trimmed to 75 bases) were deposited in the Sequence Read Archive (BioProject, PRJDB37895; Run: DRR791415‐DRR791446). Using the SNP genotype data, principal component analysis (PCA) was performed for individual clustering using Adegenet version 2.1.10 (Jombart, 2008).

Among the 39 mtDNA sequences, there were 59 variable sites, 35 of which were parsimony‐informative (24 singleton sites). The MP tree in Figure 1b showed that BE‐454 belongs to a clade consisting of only *T. orientalis*. One of the 15 *T. thynnus* species formed a clade with *T. alalunga*, most likely because of introgression. The MP tree identified BE‐454 as *T. orientalis*, whereas the possibility of *T. thynnus* persisted due to ILS.

After a series of nuclear SNP filtering, 4458 SNPs were used for the PCA. The first and second principal component scores (PC1 and PC2, respectively) are shown in Figure 1c. The PC1 scores showed a clear separation between the reference specimens of *T. orientalis* and *T. thynnus*, whereas PC2 characterized the polymorphisms within the species. The PC1 score of BE‐454 was very similar to that of *T. orientalis* and substantially different from *T. thynnus*. The nDNA analysis also suggested that BE‐454 belongs to *T. orientalis*.

In this thorough examination of external morphology, mtDNA phylogeny and nDNA genotyping, BE‐454 was conclusively determined to be *T. orientalis*, even though it was found in the Atlantic Ocean. *T. orientalis* is capable of migrating across the Pacific Ocean. The general migration patterns and life histories of this species have been investigated using electronic tagging technologies and fishery information (Fujioka et al., 2015). Some of the young *T. orientalis* born on the western side of the North Pacific move to the eastern side of the North Pacific, while the migrants return to the western Pacific after a few years (Bayliff, 1994). In addition, a small proportion of large *T. orientalis* is known to move to the southern hemisphere (Bayliff, 1994).

Owing to its high economic importance, *T. orientalis* is subject to fishing throughout its entire life stages after growing to approximately 20 cm (Nakatsuka et al., 2017), whereas almost all catch records for this species are in the Pacific. In recent years, few *T. orientalis* catches have been reported in the Indian Ocean (Di Natale et al., 2022; Zaki et al., 2017). Furthermore, a fish caught off the coast of southwestern Brazil was reported to be *T. orientalis* based on mtDNA analysis (Schroeder et al., 2025). Given the inherent issues of mtDNA barcoding in *Thunnus* as described above, it is unclear if the specimen was ‘true’ *T. orientalis*.

This study thoroughly examined the identity of a fish caught in the vicinity of Namibia and reported the first robust evidence of *T. orientalis* in the Atlantic Ocean based on morphology, mtDNA and nDNA. The Japan Fisheries Agency conducts monitoring at landing ports for catch reports for tuna species, which includes species identification using mtDNA. Although this identification is incapable of conclusively distinguishing *T. orientalis* and *T. thynnus* as described above, to date, in these catch reports, no *T. orientalis* has been found in approximately 20,000 tuna caught in the Atlantic Ocean.

## AUTHOR CONTRIBUTIONS

A.T. performed the morphological investigation. M.S. conducted the DNA analysis for this specimen. K.N. firstly found this specimen and conduct initial mitochondrial DNA analysis. N.S. supervised this study and supported the DNA analysis. Y.T. led specimen sampling and manuscript preparation. All authors interpreted the results and edited the manuscript.

## FUNDING INFORMATION

This work was supported by the Research and Assessment Program for Fisheries Resources, the Fisheries Agency of Japan.

## Supporting information


**Supporting Information 1.** PCR amplification of the mitochondrial ATP synthase subunit 6, cytochrome *c* oxidase subunit III and 16S rRNA genes.

**Supporting Information 2.** Accession numbers of mitochondrial DNA sequences.

**Supporting Information 3.** SNP calling and filtering.

## Data Availability

All mitochondrial and raw RAD‐derived sequence data that support the findings of this study will be openly available in DDBJ/EMBL/GenBank database after the acceptance of this manuscript.
