# Supplementary material for: Pacific bluefin tuna (Thunnus orientalis) bycatch in the Atlantic Ocean
Source: J Fish Biol. 2026 Apr 21;109(2):1277–81. doi: 10.1111/jfb.70463 (PMC13521704; doi:10.1111/jfb.70463)
Supplement: Supplementary file 1 [file JFB-109-1277-s001.docx]

Supporting Information 1. PCR amplification of the mitochondrial ATP synthase subunit 6, cytochrome *c* oxidase subunit III, and 16S rRNA genes

For 32 tuna specimens, we conducted PCR amplification of partial ATP synthase subunit 6 (*ATP6*), cytochrome *c* oxidase subunit III (*COX3*), and 16S rRNA (*16S*) genes using AmpliTaq Gold DNA polymerase (Thermo Fisher Scientific) combined with LifeTouch Thermal Cycler (Bioer Technology).

The PCR primers for the amplification of *ATP6* and *COX3* (ATCO primers; Chow and Kishino, 1995) are as follows:

L8562, 5'-CTTCGACCAATTTATGAGCCC-3'

H9432, 5'-GCCATATCGTAGCCCTTTTTG-3'.

According to a complete mitogenome sequence of the Pacific bluefin tuna *Thunnus orientalis* (acc., KF906721), the L8562 primer is located on the 15th to 35th positions of *ATP6* (full length: 684 bases), while the H9432 primer stretches from the 226th to 245th positions of *COX3* (full length: 785 bases). The expected PCR amplicon size with the ATCO primers is 915 bp in the genus *Thunnus*. Of note, the third base of the *ATP6* termination codon (TAA) doubles with the first base of the *COX3* initiation codon (ATG).

The PCR primers for the amplification of *16S* (16S primers) are described in Palumbi *et al.* (2002):

16sar-L, 5'-CGCCTGTTTATCAAAAACAT-3'

16sbr-H, 5'-CCGGTCTGAACTCAGATCACGT-3'.

In the mitogenome sequence above, the 16sar-L primer corresponds to the 920th‒939th positions of *16S* (full length: 1,682 bases), and the 16sbr-H primer, to the 1,515th‒1536th positions. The primer set is expected to yield 617-bp PCR amplicons according to the mitogenome sequence.

**PCR mixture (final volume 20 µl; commonly applied to ATCO and 16S primers)**

10×PCR Gold Buffer (25 mM MgCl_2_)	2.0 µl (2.5 mM MgCl_2_)

dNTPs (2 mM each)	2.0 µl (200 µM each)

Primer 1 (5 pmol/µl)	0.5 µl (125 nM)

Primer 2 (5 pmol/µl)	0.5 µl (125 nM)

Taq polymerase (5 U/µl)	0.2 µl (1.0 U)

DNA (> 4.0 ng/µl)	1.0 µl

Nuclease-free sterilized water	13.8 µl

**PCR cycling (touchdown PCR)**

The basal annealing temperature (*T*_a_) in touchdown PCR for the ATCO primers was set at 52℃, and for the 16S primers, at 50℃.

94℃	11.5 min (preheating)

94℃	15 sec

*T*_a_ + 10℃	30 sec	10 cycles (decreasing 1℃ per cycle)

72℃	1 min

94℃	15 sec

*T*_a_℃	30 sec	35 cycles

72℃	1 min

72℃	5 min (final elongation)

10℃	Hold

**References**

Chow, S., & Kishino, H. (1995). Phylogenetic relationships between tuna species of the genus *Thunnus* (Scombridae: Teleostei): Inconsistent implications from morphology, nuclear and mitochondrial genomes. *Journal of Molecular Evolution*, 41, 741‒748.

Palumbi, S., Martin, A., Romano, S., McMillan, W.O., Stice, L., & Grabowski, G .(2002). The simple fool's guide to PCR version 2.0. Department of Zoology and Kewalo Marine Laboratory, University of Hawaii.

(<https://stacks.stanford.edu/file/druid:yh393jm6703/Simple_Fool%27s_Master%20PCR.pdf>; last accessed on 6 October, 2025).
