# Supplementary material for: Pacific bluefin tuna (Thunnus orientalis) bycatch in the Atlantic Ocean
Source: J Fish Biol. 2026 Apr 21;109(2):1277–81. doi: 10.1111/jfb.70463 (PMC13521704; doi:10.1111/jfb.70463)
Supplement: Supplementary file 2 [file JFB-109-1277-s002.docx]

Supporting Information 3. SNP calling and filtering

**Short-read mapping onto a reference genome sequence**

We used the subprogram *process_radtags* in Stacks version 2.65 (Catchen *et al.,* 2011) to demultiplex the raw RAD-derived short reads and trim them to 75 bases. The reads were then quality-filtered, *i.e*., reads with a base quality < 20 at four or more bases were discarded (FASTX-Toolkit, https://github.com/agordon/fastx_toolkit; last accessed on 8 October, 2025). Using bwa-mem2 version 2.2.1 (Vasimuddin *et al*., 2019), we mapped the clean reads onto a chromosome-level reference genome sequence of the Atlantic bluefin tuna *Thunnus thynnus* (GCF_963924715.1). The number of *Sbf* I-recognition sites in the reference is 22,810. During mapping, chimeric reads (SA tag) and multiple-hit reads (XA tag) were removed.

**SNP calling**

After the short-read mapping, the resulting bam files were converted to the mpileup format using the subprogram *mpileup* in SAMtools version 1.13 (Li *et al*., 2009), in which we set the minimum mapping quality and base quality at 40 and 30, respectively. Based on the mpileup file, SNPs were called with the subprogram *mpileup2snp* in VarScan2 version 2.4.4 (Koboldt *et al*., 2012). The parameters defined in this computation were as follows: --min-coverage 30, --min-freq-for-hom 0.80, --min-avg-qual 30, --min-read2 7, --min-var-freq 0.05, and --p-value 0.05.

**SNP filtering**

In the following SNP filtering, we used VCFtools version 0.1.6 (Danecek *et al*., 2011), unless otherwise specified. The specimens (*N* = 32) were divided into a Pacific bluefin *Thunnus orientalis* population (abbreviation, PBF; *N* = 16), Atlantic bluefin population (ABF; *N* = 15), and an undetermined specimen “BE-454”.

We skipped coverage-depth filtering as this was already done in the precedent SNP calling (*i.e*., --min-coverage 30 in VarScan2). Among the called SNPs, biallelic SNPs were retained. SNPs with a proportion of missing genotypes (missingness) > 0.05 across the specimens were omitted (max-missing filtering). After SNPs with a minor allele frequency of ≥ 0.05 in the whole specimens were selected (the resulting VCF file is called “MAF.vcf” henceforth), we applied the max-missing filtering to each of ABF and PBF in the MAF.vcf using a bash script (pop_missing_filter.sh) available in the dDocent website (<https://ddocent.com/>; last accessed on 8 October, 2025). No missing genotype was found in ABF and PBF after this filtering step. We further excluded SNPs that were out of Hardy-Weinberg equilibrium in either ABF or PBF, or both (*P* < 0.05) using a perl script (filter_hwe_by_pop.pl; obtained from the dDocent website). SNP sites in the resulting VCF file were listed, and genotype data of these sites were extracted from the MAF.vcf. Finally, we pruned SNPs so that two SNPs in a chromosome (scaffold) was at least 100k bases apart, following the method as described in the Supporting Information S4 of Sekino *et al.* (2023). We did not employ filtering based on linkage disequilibrium because of the small sample sizes of ABF and PBF.

As a result of these filtering steps, 4,458 SNPs were retained. All specimens excluding BE-454 had no missing genotype; BE-454 had merely one missing genotype. We considered that these SNPs are widely scattered across the genome, as there was a positive linear correlation between the length of chromosome and the number of SNPs (Fig. S1; *r* = 0.936).

**Figure S1.** Relationships between the number of SNPs and chromosome length

The reference genome sequence has 24 chromosomes, one unlocalized scaffold within Chromosome 2, and 94 unplaced scaffolds. The plot exclusively represents the number of SNPs in each of the 24 chromosomes. There were three SNPs in three unplaced scaffolds (SCAFFOLD_80 with the length of 57,571 bases; SCAFFOLD_95, 3,747 bases; and SCAFFOLD_106, 10,663 bases).

**References**

Catchen, J.M., Amores, A., Hohenlohe, P., Cresko, W., & Postlethwait, J.H. (2011). Stacks: building and genotyping loci de novo from short-read sequences. *G3 Genes*|*Genomes*|*Genetics*, 1, 171–182.

Danecek, P., Auton, A., Abecasis, G., Albers, C.A., Banks, E., DePristo, M.A., Handsaker, R.E., Lunter, G., Marth, G.T., Sherry, S.T., McVean, G., Durbin, R., & 1000 Genomes Project Analysis Group (2011). The variant call format and VCFtools. *Bioinformatics*, 27, 2156–2158.

Koboldt, D.C., Zhang, Q., Larson, D.E., Shen, D., McLellan, M.D., Lin, L., Miller, C.A., Mardis, E.R., Ding, L., & Wilson, R.K. (2012). VarScan2: Somatic mutation and copy number alteration discovery in cancer by exome sequencing. *Genome Research*, 22, 568–576.

Li, H., Handsaker, B., Wysoker, A., Fennell, T., Ruan, J., Homer, N., Marth, G., Abecasis, G., Durbin, R., 1000 Genome Project Data Processing Subgroup (2009). The sequence alignment/map (SAM) format and SAMtools. *Bioinformatics*, 25, 2078–2079.

Sekino, M., Hashimoto, K., Nakamichi, R., Yamamoto, M., Fujinami, Y., Sasaki, T. (2023). Introgressive hybridization in the west Pacific pen shells (genus *Atrina*): Restricted interspecies gene flow within the genome. *Molecular Ecology*, 32, 2945–2963.

Vasimuddin, Md., Misra, H., Li, H., & Aluru, S. (2019). Efficient architecture-aware acceleration of BWA-MEM for multicore systems. *IEEE Parallel and Distributed Processing Symposium*, Rio de Janeiro, Brazil. pp. 314-324.
